# Gelation and Plugging Performance of Low-Concentration Partially Hydrolyzed Polyacrylamide/Polyethyleneimine System at Moderate Temperature and in Fractured Low-Permeability Reservoir

**DOI:** 10.3390/polym16111585

**Published:** 2024-06-03

**Authors:** Kai Wang, Mingliang Luo, Mingzhong Li, Xiaoyu Gu, Xu Li, Qiao Fan, Chunsheng Pu, Liangliang Wang

**Affiliations:** 1 School of Petroleum Engineering, China University of Petroleum (East China), Qingdao 266580, China;13689288083@163.com (K.W.); limingzhong_upc@hotmail.com (M.L.); 17685539062@163.com (Q.F.); chshpu_tx@126.com (C.P.); llwang2017@163.com (L.W.); 2 College of Petroleum Engineering, Xi’an Shiyou University, Xi’an 710065, China; 190108@xsyu.edu.cn; 3 School of Vehicle and Energy, Yanshan University, Qinhuangdao 066004, China; shidalx@163.com

**Keywords:** HPAM/PEI gel, low concentration, gelation, plugging, low-permeability reservoir

## Abstract

HPAM/PEI gel is a promising material for conformance control in hydrocarbon reservoirs. However, its use in low-permeability reservoirs is limited by the high polymer concentrations present. In this study, the gelation performance of an HPAM/PEI system with HPAM < 2.0 wt.% was systematically investigated. The gelation time for HPAM concentrations ranging from 0.4 to 2.0 wt.% varied from less than 1 h to 23 days, with the highest gel strength identified as grade H. The hydrodynamic radius manifested the primary effect of HPAM on the gelation performance. Branched PEI provided superior gelation performance over linear PEI, and the gelation performance was only affected when the molecular weight of the PEI varied significantly. The optimal number ratio of the PEI-provided imine groups and the HPAM-provided carboxylic acid functional groups was approximately 1.6:1~5:1. Regarding the reservoir conditions, the temperature had a crucial effect on the hydrodynamic radius of HPAM. Salts delayed the gelation process, and the order of ionic influence was Ca^2+^ > Na^+^ > K^+^. The pH controlled the crosslinking reaction, primarily due to the protonation degree of PEI and the hydrolysis degree of HPAM, and the most suitable pH was approximately 10.5. Plugging experiments based on a through-type fracture showed that multi-slug plugging could significantly improve the plugging performance of the system, being favorable for its application in fractured low-permeability reservoirs.

## 1. Introduction

Low-permeability reservoirs, characterized by low matrix permeability and insufficient natural energy, rely heavily on water injection to maintain efficient development [1,2]. However, long-term water injection often leads to excessive water production, resulting in a range of concerns related to the environment, corrosion, and scale formation. Especially in fractured low-permeability reservoirs, under water channeling, it becomes challenging to remove the hydrocarbons surrounding the flooding zone, severely limiting the hydrocarbon recovery efficiency [3].

An effective solution to mitigate or eliminate this problem is polymer gel treatment [4,5,6,7]. Polymer gels can be categorized into inorganic crosslinked gels and organic crosslinked gels based on the type of crosslinker employed [8,9]. Inorganic crosslinkers, such as chromium and zirconium ions, crosslink with polymers through ionic and ligand bonds [10]. Organic crosslinkers, including phenol/formaldehyde systems and polyethyleneimine (PEI), crosslink with polymers through covalent bonds [11]. In recent years, the use of metal ions and phenol/formaldehyde crosslinkers has been gradually restricted in some regions due to their inherent toxicity and potential environmental risks. PEI, a low-toxicity, environmentally friendly material that is approved for use in food contact packaging in North America, has emerged as a promising crosslinker [12]. PEI can effectively crosslink polymers containing acrylamide groups, such as partially hydrolyzed polyacrylamide (HPAM) and polyacrylamide tertiary butyl acrylate (PAtBA). PAtBA, with its relatively low molecular weight (250–500 × 10^3^ Da), can form “ring gels” with PEI and exhibit high long-term stability at high temperatures (>80 °C) [13,14,15,16]. However, the complex synthesis process and high cost of PAtBA have limited the widespread adoption of PAtBA/PEI systems. This has led to the exploration of the substitution of PAtBA with the more affordable and readily available HPAM. Allison was the first to report that HPAM and PEI could form hydrogels at room temperature [17]. Jia et al. evaluated the gelation performance of an HPAM/PEI system at 40 °C and found that it exhibited a longer gelation time compared to an HPAM/CrAc (chromium acetate) system [18]. In a study by Al-Muntasheri, the HPAM/PEI system formed a strong and stable gel at 130 °C and achieved a 100% reduction in permeability under pressure gradients of 1000 psi for 3 weeks [19,20]. Compared to PAtBA/PEI or other systems that use metal ions as crosslinkers, the HPAM/PEI gel system has relatively larger polymer and crosslinker molecules. This makes the HPAM/PEI gel effective in inhibiting water channeling in fractures and high-permeability channels [21,22,23]. However, the most successful applications of PEI crosslinked polymer gel systems have been observed in extreme conditions such as high temperatures and severe formation leakages, which require high polymer concentrations (2.0–7.0 wt.%) [24,25,26,27]. High polymer concentrations lead to the high viscosity of the gelant, resulting in exceptionally high injection pressures in field tests and limiting access to deep reservoirs, especially in low-permeability reservoirs [28,29]. Meanwhile, the high cost associated with high polymer concentrations is also an important reason limiting their widespread application.

The objective of this study was to thoroughly understand the gelation performance of a low-concentration HPAM/PEI gel system at a moderate temperature and evaluate its potential for fracture plugging in fractured low-permeability reservoirs. The gelation performance of the low-concentration HPAM/PEI system (HPAM < 2.0 wt.%) was systematically investigated at 20–80 °C. The effects of the concentration and molecular structure of HPAM and PEI, as well as the temperature, salts, and pH, on the gelation performance of the low-concentration HPAM/PEI system were investigated by combining viscosity measurements, a bottle strength test, and microphotography. The structure considered here included the molecular weight and hydrolysis degree of HPAM and the molecular weight and branching degree of PEI. The plugging performance of the systems was evaluated in low-permeability cores with through-type fractures, which represent the most hazardous flood channels in low-permeability reservoirs.

## 2. Materials and Methods

### 2.1. Materials

Five types of HPAM with molecular weights of 5, 7, 10, 12, and 14 × 10^6^ Da were used, all in dry powder form with an active content of over 98%. Linear PEI (L-PEI) was received as a powder with a molecular weight of 25,000 Da. Four types of branched PEI (B-PEI) with molecular weights of 600, 1800, 10,000, and 25,000 Da were received as liquids. The salts used to prepare the brine included sodium chloride (NaCl), potassium chloride (KCl), magnesium chloride (MgCl_2_), and calcium chloride (CaCl_2_). Deuterium oxide (D_2_O) was used as a solvent for the NMR tests. A methyl orange solution (1 g·L^−1^) and indigo carmine (96%) were used for the HPAM hydrolysis titration test. All of the above were purchased from Macklin Inc. (Shanghai, China). Solid sodium hydroxide (NaOH) and hydrochloric acid (HCl) (37%) were purchased from Sinopharm (Shanghai, China).

### 2.2. HPAM Hydrolysis Degree Test

To analyze the effect of the HPAM hydrolysis degree on the gel performance, a solution of 0.03 wt.% HPAM in deionized water (100 mL) was prepared. The HCl solution was diluted to a concentration of 0.1 mol·L^−1^. Then, 0.1 wt.% methyl orange and 0.25 wt.% indigo carmine were each placed into two burettes to serve as indicators. The test was initiated by adding one drop of each indicator to the HPAM solution, resulting in a yellowish green hue. Titration was then begun with the 0.1 mol·L^−1^ HCl, continuing until the solution transitioned from yellowish green to light gray, signifying the end point of the reaction. The test was carried out at room temperature, at least 3 measurements were taken for each sample, and the results were averaged. The hydrolysis degree of HPAM was calculated via Equation (1).

*HD* = (*c*·*V* × 71 × 100)/(1060 *m*·*s* − 23 *c*·*V*) (1)

where *HD* is the hydrolysis degree of HPAM, %; *c* is the concentration of hydrochloric acid solution, mol·L^−1^; *V* is the volume of the hydrochloric acid solution consumed by the HPAM solution, mL; *m* is the mass of HPAM, g; *s* is the effective content of HPAM, %; 23 is the difference between the relative mass of the carboxyl group and that of the acrylamide group; and 71 is the relative mass of acrylamide links corresponding to 1.00 mL of the hydrochloric acid solution.

### 2.3. B-PEI Branching Degree (BD) Test

To analyze the effect of the PEI branching degree on the gelation performance, B-PEI solutions were prepared in D_2_O at a concentration of 25 mg·mL^−1^. The ^13^C-NMR spectra were acquired for these solutions at 25 °C using a JNM-ECZ400S spectrometer (JEOL from Beijing, China). Eight prominent peaks were seen in the ^13^C-NMR spectrum of B-PEI. These peaks included those at 56.1, 53.1, and 51.1 ppm, which corresponded to methylene groups within the tertiary amine units. Additionally, the peaks at 50.7, 47.7, and 45.7 ppm were indicative of secondary amines, while those at 39.8 ppm and 37.8 ppm were indicative of methylene groups within the primary amines. The branching degree of B-PEI was determined using Equation (2) [30]:

*BD* = 2*D*/(2*D* + *L*) (2)

where *D* is the peak area corresponding to the tertiary amine branched units and *L* is the sum of the peak areas corresponding to the primary and secondary amine linear units. The individual absorption peak areas were automatically recognized and calculated using the MestReNova LITE software (9.0.1).

### 2.4. Hydrodynamic Radius (R_h_) Test of HPAM

Dynamic light scattering is a reliable tool for obtaining the stretching state of HPAM’s molecular chains in a solution [31,32]. The principle of this method is to analyze the short-term fluctuations of scattered light, which allows the measurement of the Brownian motion velocity and particle size using the Stokes–Einstein equation [33]. The hydrodynamic radius (*R_h_*) of HPAM in the solution (0.03 wt.%) was determined using a Malvern Zetasizer Lab analyzer equipped with a dynamic light scattering (DLS) module. The measurements were taken at a fixed scattering angle of 90° and a wavelength of 532 nm, with a minimum of 3 measurements for each sample, and the results were averaged. The hydrodynamic radius (*R_h_*) was obtained from the DLS software (1.0.0.1).

### 2.5. Preparation of HPAM/PEI Gel

The HPAM solution was prepared by gently adding a predetermined amount of HPAM to stirred deionized water or brine. The stirring rate was adjusted so that the vortex reached approximately 2/3 of the distance from the top surface to the propeller. The HPAM solution was stirred at 600 rpm for 1 h and then at a speed of 400 rpm for at least 2 h, and it was aged for 24 h. The PEI solution was added to the HPAM solution with continuous stirring for 10 min to obtain the gelant.

The crosslinking of HPAM with PEI is primarily due to the electrostatic interaction between the primary and secondary amines of PEI with the carboxylic acid functional groups in HPAM.

### 2.6. Gel Performance Evaluation

#### 2.6.1. Viscosity Measurement

**Figure 1:**
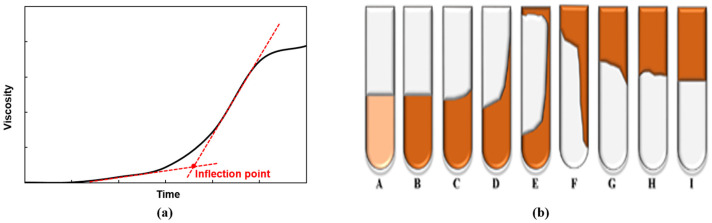
(**a**) Inflection point on viscosity versus time curve and (**b**) gel strength code reference chart.

To obtain the gelation time, the relationship between the viscosity and time for the HPAM/PEI gel was measured under constant shear rate conditions (2.04 s^−1^) using a Brookfield DV-III rotational viscometer at 6 rpm with a No. 64 spindle. Moreover, viscosity measurements were taken periodically to obtain the viscosity versus time curve until the viscosity of the gel exceeded the maximum limit (12,000 mPa·s) of the viscometer. The time required to reach the inflection point on the viscosity versus time curve was defined as the gelation time (Figure 1a), indicating the onset of gelation.

#### 2.6.2. Bottle Test Method

**Figure 2:**
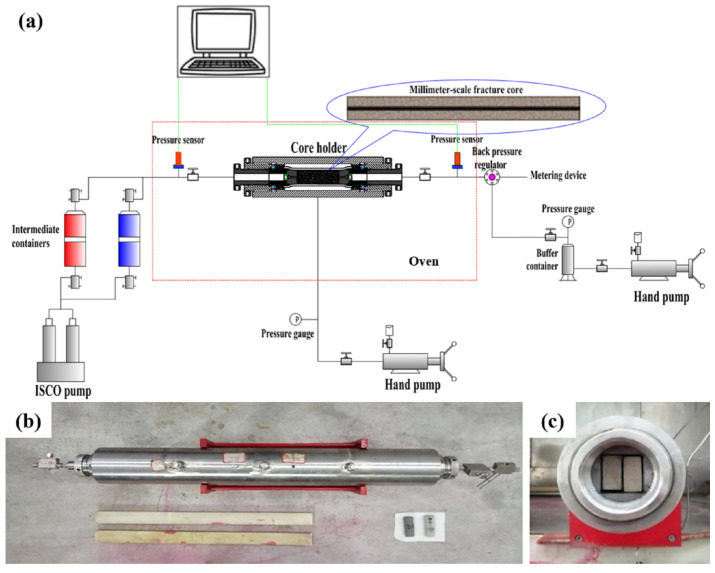
(**a**) Experimental setup for the plugging performance test; (**b**) fracture core holder; and (**c**) side view of core holder end.

The bottle test method is a practical and time-efficient semi-quantitative approach to evaluating gel strength. In our study, this was performed by transferring 30 mL of the prepared gel solution into 50 mL vials, which were then placed in an oven. At certain intervals, the vials were taken out and inverted for visual inspection, and an appropriate code indicating the gel strength was assigned [34]; see Figure 2b. For the viscosity test and the strength test of the gels, the same three copies of each gel sample were prepared to ensure the reproducibility of the results.

#### 2.6.3. Microstructure of Mature Gel

The Alpha 2–4 plus freeze dryer was used to rapidly freeze the mature gel, and the water was drained to obtain freeze-dried samples of the matured gels. A few freeze-dried samples were taken and placed on an observation bowl for conductive spraying. The microstructures of these samples were examined using an S4800 cold field emission scanning electron microscope (SEM, Hitachi from Tokyo, Japan).

### 2.7. Fractured Core Plugging Testing

The fractured core plugging test was used to evaluate the performance of the gel system in plugging a through-type flood channel. Artificial cores with a length of 60 cm and a cross-section of 4.5 × 4.5 cm were split axially and thin steel wires were clamped into the split cores to obtain fractured cores. Before fracturing, the primary permeability of all of the cores to brine was less than 5 × 10^−3^ μm^2^, and the threshold pressure gradient was approximately 470 kPa·m^−1^. The threshold pressure gradient is the minimum driving pressure required for a fluid to flow in a porous medium. If the displacement pressure gradient is greater than the threshold pressure gradient, the injected water will enter the matrix to move out the oil. The fractured cores with openings of 2.0 mm were filled with sand and cast with epoxy resin. The density of sand filling in the fracture was approximately 50 kg·m^−2^, the fracture volume after sand filling was approximately 28 mL with a porosity of 0.52, and the permeability of the fractured core was approximately 15 μm^2^. The experimental setup and the fractured core used are shown in Figure 2.

Experimental process:

1. The testing of the tightness of the equipment and the initial permeability of the cores was performed following our previous work [35].
2. Gelant injection: The gelant was injected into the cores immediately after preparation at 1 mL·min^−1^; then, the core holder was closed for a period of time to allow the gel to mature. When injecting a gel into multiple slugs, the next slug should be injected only after the previous gel has matured. In this study, after each slug of gel was injected, the pipeline was cleaned with brine immediately to prevent the pipeline from becoming blocked.
3. Evaluation of the plugging performance: After all the injected gel had matured, brine was injected to determine the gel plugging performance.

## 3. Results and Discussion

### 3.1. Effect of HAPM and PEI on Gelation Performance

#### 3.1.1. Effect of HPAM Structure

The gelation performance of HPAM with different structural parameters was evaluated with a concentration of 2.0 wt.% at 40 and 60 °C. The molecular weight of the B-PEI was 25,000 Da and the concentration was 0.2 wt.%.

**Figure 3:**
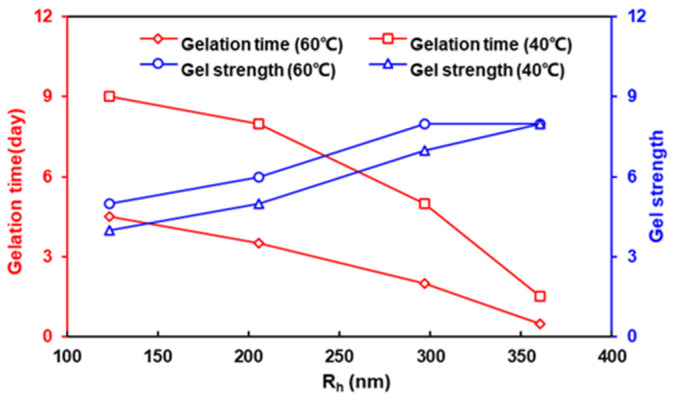
Effect of hydrodynamic radius of HPAM on gelation time and gel strength.

It is clear that the gelation rate is directly and positively correlated with the gel strength; see Table 1. However, since the HPAMs varied in both molecular weight and hydrolytic degree, the direct relationship between the gelation time and gel strength and the molecular weight or hydrolytic degree was not clear. In view of this, we introduced the hydrodynamic radius, the molecular structure of HPAM in the solution state. Moreover, its effect on the gelation time and gel strength were analyzed (Figure 3). The gel strength codes (A–I) of the bottle test were converted into numerical values (1–9). The results showed that there was a noticeable upward trend in both the gelation rate and gel strength as the hydrodynamic radius of the HPAM increased. In particular, the HPAM with the largest hydrodynamic radius (Mw = 12 × 10^6^ Da, *HD* = 7.64%) exhibited the shortest gelation time (0.5 day) and the strongest gel (H grade). Conversely, the HPAM with the smallest hydrodynamic radius (Mw = 14 × 10^6^ Da, *HD* = 3.75%) failed to gel within 30 days at both 40 °C and 60 °C. This indicates that the hydrodynamic radius is the primary driver of the effect of HPAM on the gelation performance. Moreover, generally, larger molecules of HPAM should correspond to a larger hydrodynamic radius, resulting in better gelation performance. However, our experimental results seem to contradict this rule. For instance, the 5 × 10^6^ Da HPAM (*HD* = 9.62%) had a hydrodynamic radius of 297.1 nm at 60 °C, surpassing even the 14 × 10^6^ Da HPAM (*HD* = 3.75%), owing to its notably high hydrolysis degree (9.6%). On the other hand, the 12 × 10^6^ Da HPAM (*HD* = 7.64%) displayed the shortest gelation time and the highest strength, corresponding to the fact that it possessed the largest hydrodynamic radius of 360.3 nm. It is evident that the hydrodynamic radius of HPAM in a solution is not only determined by its molecular weight but is closely related to its hydrolysis degree.

Since we were not able to obtain a series of HPAMs with identical molecular weights and varying degrees of hydrolysis and/or a series of HPAMs with identical degrees of hydrolysis and varying molecular weights, the dependence of the hydrodynamic radius on the molecular parameters was not systematically investigated. Nevertheless, we can derive some laws from the theoretical analysis of the influence of the molecular weight and hydrolysis degree on the hydrodynamic radius and gelation performance. The molecular weight, or the number of repeating units in a single polymer chain, directly determines the initial size of the HPAM molecules, and the hydrolysis degree influences the stretched state of the HPAM chains in the solution because the higher the hydrolysis degree, the more negatively charged carboxyl groups are present on the HPAM chain; then, the increased electrostatic repulsion due to the carboxyl groups will result in a larger hydrodynamic radius [14]. In addition, a more stretched HPAM chain exposes more amide groups as potential crosslinking sites [36]. Moreover, the increased carboxyl groups from hydrolysis can attract PEI with protonated amines in close proximity to HPAM molecules. However, it is important to note that a higher hydrolysis degree does not always result in improved gelation. A higher hydrolysis degree reduces the number of amide groups available for crosslinking. Therefore, for a given molecular weight of HPAM, the hydrolysis degree should ideally be optimized to achieve the better gelation performance of the HPAM/PEI system.

#### 3.1.2. Effect of PEI Structure

The structure of PEI includes its molecular weight and branching degree, with the branching degree of L-PEI designated as zero. The mass concentrations were 2.0 wt.% for HPAM (12 × 10^6^ Da) and 0.2 wt.% for PEI. The specific parameters for PEI are shown in Table 2.

**Figure 4:**
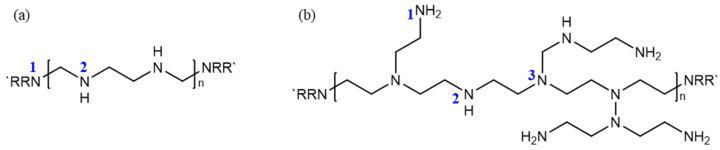
Structural diagram of PEI (25,000 Da): (**a**) linear PEI; (**b**) branched PEI (1—primary amine, 2—secondary amine, 3—tertiary amine).

The results clearly demonstrate that the gelation performance of L-PEI significantly lags behind that of B-PEI. The carboxylic acid functional groups in HPAM can crosslink with both the primary and secondary amines on PEI, with a preference for crosslinking with the more active primary amines. B-PEI molecules feature a substantial number of primary amines in their repeating units, while L-PEI molecules contain significantly fewer reactive secondary amines in their repeating units, with primary amines present only at the chain ends; see Figure 4. Table 3 shows the relative mass ratios of nitrogen for each of the three types of amino groups in L-PEI and B-PEI with a molecular weight of 25,000 Da. The relative mass ratio of primary-amine nitrogen in B-PEI is over 120 times higher than that in L-PEI; specifically, 13.55% primary-amine nitrogen in B-PEI, compared to only 0.11% in L-PEI. This stark contrast is the fundamental reason that B-PEI exhibits superior performance in the context of the HPAM/PEI system. The following experiments and discussions in our work focused on B-PEI.

Theoretically, as the molecular weight of B-PEI increases and the degree of branching decreases, the gelation time for HPAM/PEI should decrease while the gel strength increases. First, higher-molecular-weight PEI reduces the distance between itself and the HPAM, and exposes more reactive primary amine groups for crosslinking [30]. Second, according to Equation (2), a lower branching degree results in a higher percentage of primary amines. In our experiments, reducing the branching degree of B-PEI did not appear to significantly improve the gelation performance, even though the molecular weight of the PEI increased at the same time. For instance, when the branching degree was decreased from 52% (600 Da) to 45% (1800 Da), there was no significant change in the gelation time or gel strength. By contrast, PEI with a molecular weight of 10,000 Da displayed an accelerated gelation time of one day compared to PEI with a molecular weight of 1800 Da at 60 °C. Moreover, the gelation time was shortened by 1.5 days, and the gel strength improved from grade G to H at 40 °C. In other words, the macroscopic gelation performance of the HPAM/PEI system only emerged when the structure of the PEI, especially the molecular weight, changed dramatically.

#### 3.1.3. Effect of HPAM Concentration

B-PEI (25,000 Da) was used at a fixed concentration of 0.2 wt.%. The concentrations of HPAM (Mw = 12 × 10^6^ Da) varied from 0.4 wt.% to 2.0 wt.%.

First, the initial viscosity of the system increased significantly with the increasing HPAM concentration, as shown in Table 4. The initial viscosity of the system at HPAM concentrations above 1.2 wt.% was greater than 100 mPa·s, while the initial viscosity of most gel systems used in low-permeability reservoirs is approximately 20~60 mPa·s [37]. The high viscosity will undoubtedly render it difficult to inject the gelant into low-permeability reservoirs.

**Figure 5:**
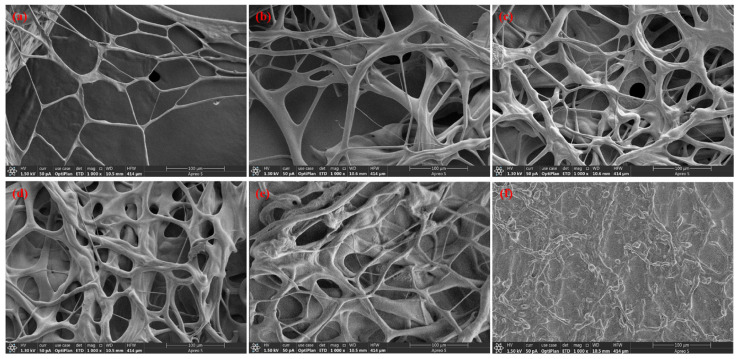
Microstructures of matured gels with different HPAM concentrations: (**a**) 0.4 wt.%; (**b**) 0.8 wt.%; (**c**) 1.2 wt.%; (**d**) 1.6 wt.%, (**e**) 2.0 wt.% and (**f**) 0.4 wt.% HPAM solution.

On the other hand, as the polymer concentration increased, the gelation time decreased from 3 days to 0.5 days at 60 °C and from 4 days to 1 day at 40 °C. Simultaneously, the gel strength improved from grade D to grade H at 60 °C and from grade D to grade G at 40 °C. However, beyond a HPAM concentration of 1.2 wt.%, the gel strength did not increase further, and only the gelation time continued to decrease at both 40 °C and 60 °C. The microstructures of the matured HPAM/PEI gels (Figure 5) also exhibited this trend. The three-dimensional network of the matured gels showed a denser network and smaller network spacing as the HPAM concentration was increased from 0.4 wt.% to 1.2 wt.%. A further increase in the HPAM concentration from 1.6 wt.% to 2.0 wt.% did not result in a significant change in the network structure. The higher concentration of HPAM provided more carboxylic acid functional groups for crosslinking, which promoted faster gelation and increased strength. However, the ratio of HPAM to PEI should not be too high; if this occurs, PEI will become the limiting reactant. Therefore, further increasing the HPAM concentration will not significantly improve the gelation performance.

**Figure 6:**
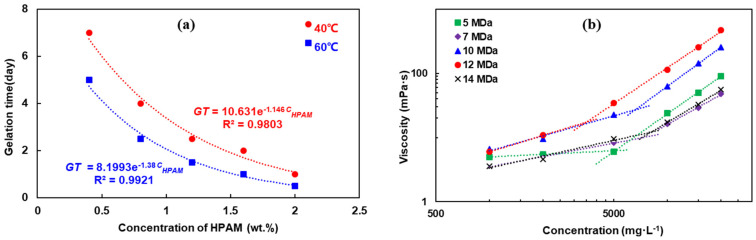
(**a**) HPAM concentration versus gelation time; (**b**) concentration versus apparent viscosity of HPAM solution.

The relationship between the HPAM concentration (0.4–1.2 wt.%) and gelation time was further investigated. As shown in Figure 6a, there exists a clear exponential relationship between the gelation time and HPAM concentration, at both 60 °C and 40 °C, where *C_HPAM_* represents the HPAM concentration and *GT* is the gelation time. This observation is consistent with the findings of Jia (40 °C, *C*_PEI_ = 1.0 wt.%), Al-Muntasheri (120 °C, *C*_PEI_ = 0.3 wt.%), and Hardy (96 °C, *C*_PEI_ = 2.0 wt.%) [18,20,32]. In a study by Jia, the gel stability decreased when the HPAM concentration fell below 1.0 wt.%. Additionally, the gel failed to achieve gelation when the HPAM concentration was below 0.5 wt.%, regardless of the PEI concentration. Therefore, this study identified 0.5 wt.% as the minimum critical concentration of HPAM, while in our experiments, a grade D gel was still formed with 0.4 wt.% HPAM and 0.2 wt.% PEI.

The HPAM molecules in a solution exhibit a random arrangement resembling a mass of spherical coils. As the polymer concentration increases, the distance between these molecules gradually decreases until they overlap and intertwine. The intermolecular crosslinking reaction occurs when the functional groups of the polymer chains approach an effective distance. The concentration at which the polymer molecules begin to overlap and interpenetrate is known as the critical overlap concentration (C*). In polymer gel systems, the polymer concentration must exceed this critical overlap concentration to achieve a continuous three-dimensional microstructure in the gel [28]. The critical overlap concentration can be determined from the logarithmic relationship between the polymer concentration and apparent viscosity. The polymer viscosity exhibits a two-stage linear change with the concentration, and the inflection point between these two stages represents the critical overlap concentration [38]. Figure 6b illustrates the relationship between the HPAM concentration and apparent viscosity on a double-logarithmic scale, yielding the critical overlap concentration, as summarized in Table 5.

There is a negative correlation between the critical overlap concentration of HPAM and its hydrodynamic radius. More directly, the critical overlap concentration is also determined by the molecular weight and the hydrolysis degree of the HPAM. For instance, due to the high hydrolysis degree, HPAM with a molecular weight of 12 × 10^6^ Da had a lower critical overlap concentration than HPAM with a molecular weight of 14 × 10^6^ Da, corresponding to 3836.95 mg·L^−1^ (about 0.38 wt.%). This is also the reason that it can still gel at a low concentration of 0.4 wt.%.

#### 3.1.4. Effect of PEI Concentration

The concentration of HPAM (12 × 10^6^ Da) was maintained at 1.0 wt.%, and the effect of the PEI (branched, 25,000 Da and 1800 Da) concentration (0.05–0.6 wt.%) on the gelation time and gel strength at 60 °C was investigated. The results are summarized in Table 6. It is worth mentioning that with the HPAM concentration set at 1.0 wt.%, the initial viscosity of the gelant essentially remained stable at around 114 mPa·s at 60 °C, regardless of the change in the PEI concentration. In other words, the initial viscosity of the system was mainly determined by the concentration of HPAM.

In general, the gelation time tended to decrease with the increasing PEI concentration, while changes in the gel strength appeared to be sluggish. It appears that the concentration of HPAM is the primary determinant of the gel strength. Moreover, when the PEI concentration exceeded 0.4 wt.%, HPAM became the limiting reactant for the crosslinking reaction, resulting in no significant change in the gelation time and gel strength. Furthermore, when the PEI concentration dropped below 0.05 wt.%, the PEI with a molecular weight of 1800 Da was unable to induce gelation, whereas the PEI with a molecular weight of 25,000 Da achieved a gel strength of grade D. This suggests that the minimum critical concentration of PEI can be reduced by increasing the molecular weight.

An excessive ratio of ionic crosslinker to polymer can lead to over-crosslinking phenomena such as flocculation and precipitation, and previous studies have indicated that the critical crosslinking ratio of PEI to acrylamide-based polymers is 1:2 [39]. However, in our experiments, no over-crosslinking was observed, even though the crosslinking ratio of PEI to HPAM was 3:5. We believe that the crosslink between HPAM and PEI stops when one of the reactants is depleted. Considering the results of the HPAM concentration analysis, we suggest that the appropriate mass concentration ratio of PEI to HPAM is within an approximate range of 2:5 to 2:16. Beyond this range, one of the reactants becomes a limiting reactant and the other is wasted due to overload. Additionally, based on the experimental conditions and their molecular structures, the ratio of the number of PEI-provided imine groups and HPAM-provided carboxylic acid functional groups is approximately 1.6:1~5:1. This result may provide guidance for future studies or applications of the HPAM/PEI gel system in the selection of HPAM and PEI.

**Figure 7:**
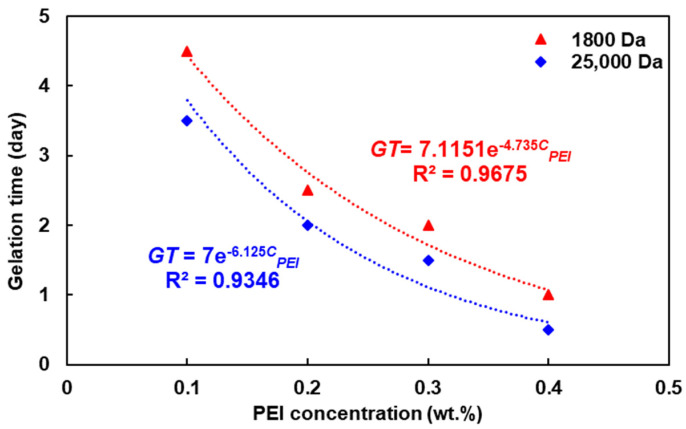
PEI concentration (0.1–0.4 wt.%) versus gelation time of HPAM/PEI gel system.

The curve depicting the PEI concentration versus the gelation time reveals an exponential decrease in the gelation time with the increasing PEI concentration; see Figure 7. A comparison between the concentration versus gelation time fitting equations for HPAM and PEI shows that the correlation coefficient between the HPAM concentration and gelation time is higher (Figure 6a and Figure 7), indicating that changes in the HPAM concentration exert a more significant influence on the gelation time. Table 4 and Table 6 also show that the effect of changing the HPAM concentration on the gel strength is more pronounced than that of PEI. In the HPAM/PEI gel system, due to its much higher molecular weight and much larger hydrodynamic radius compared to PEI, HPAM is the foundation of the three-dimensional network of the mature gel [40]. HPAM thus determines the mass transfer distance of the PEI molecules in the crosslinking reaction and the network density of the mature gel. Consequently, changes in both the structure and concentration of HPAM have a more pronounced impact on the gelation performance than changes in those of PEI.

### 3.2. Effect of Reservoir Conditions on Gelation Performance

#### 3.2.1. Temperature

The gelation processes of three HPAM/PEI systems with different concentrations of HPAM and PEI were investigated at 20–80 °C, focusing on HPAM with a molecular weight of 12 × 10^6^ Da and branched PEI with a molecular weight of 25,000 Da. The results are summarized in Table 7.

**Figure 8:**
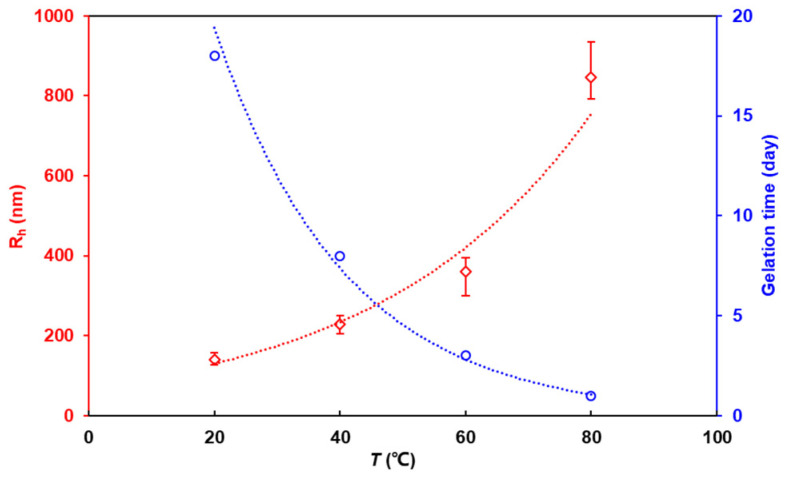
Effect of temperature on R_h_ of HPAM (12 × 10^6^ Da) and gelation time.

As the temperature increased, the gelation time decreased and the gel strength increased for all three HPAM/PEI systems. This can be attributed to several factors. First, an elevated temperature increases the thermal motion of the HPAM and PEI molecules, thereby increasing the probability of effective collision and subsequent crosslinking between them [41]. Secondly, the primary amine groups in PEI that serve as crosslinking sites become more susceptible to crosslinking as the temperature increases [42]. Furthermore, an increase in temperature promotes the hydrolysis of HPAM, as hydrolysis is a heat-absorbing process. The increase in hydrolysis resulted in more of the amide groups of HPAM being hydrolyzed to carboxylic acid groups, as well as more stretching of the molecule with a larger hydrodynamic radius (effect of electrostatic repulsion between carboxylic acid groups), leading to improved gelation performance. Specifically, the hydrodynamic radius of the HPAM (12 × 10^6^ Da, 0.03 wt.%) increased by more than six times as the temperature increased from 20 °C to 80 °C, with the largest increase occurring between 60 °C and 80 °C; see Figure 8.

To better understand the influence of the temperature on the crosslinking reaction, we introduced the Arrhenius-type equation:

*GT* = *A*·*exp*(*Ea*/*RT*) (3)

where *GT* is the gelation time, *A* is the frequency factor in min, *Ea* is the activation energy in kJ·mol^−1^, *R* is the ideal gas constant, which is taken as 8.314 J·(mol·K)^−1^, and *T* is the temperature in *K*.

The activation energy (*Ea*) quantifies the temperature sensitivity of the gelation process and reflects the ease or difficulty of the process [43]. Higher activation energy signifies a more challenging and temperature-sensitive reaction. Taking the logarithm of the Arrhenius equation gives the following expression:

*ln*(*GT*) = *lnA* + *Ea*/*RT* (4)

**Figure 9:**
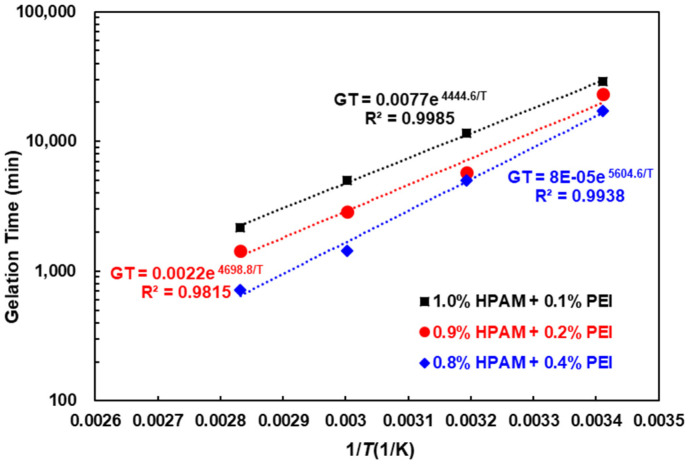
Arrhenius-type curve of gelation time versus temperature.

Plotting *ln*(*GT*) against 1/*T* yields a linear relationship with a slope of *Ea*/*R* and an intercept of *lnA*; see Figure 9. The calculated *Ea* values for the three HPAM/PEI systems were 36.95, 39.07, and 46.60 kJ·mol^−1^, respectively, with an average activation energy of 40.87 kJ·mol^−1^. These values are in close agreement with those reported by Ghriga [30].

The HPAM concentration gradually decreased by 0.1% while the PEI concentration doubled among the three HPAM/PEI systems, as shown in Table 7. At a given temperature, the gelation time decreases with an increasing PEI concentration, but the final gel strength is reduced with a decreasing HPAM concentration. For instance, at 60 °C, the gel strength of system 1 is grade G, that of system 2 is grade F, and that of system 3 is reduced to grade E. In addition, the activation energy (*Ea*) increases from system 1 to system 3, indicating a more difficult crosslinking reaction. This also emphasizes that the HPAM concentration has a more significant impact on the ease of the crosslinking reaction and the final gel strength of the HPAM/PEI system compared to PEI. Considering the effect of the temperature on the hydrodynamic radius of HPAM, it can be hypothesized that the temperature-induced improvement in the gelation performance is considerably dependent on the effect of the temperature on the structure of HPAM.

#### 3.2.2. Salt

In this experiment, HPAM with a molecular weight of 12 × 10^6^ Da and branched PEI with a molecular weight of 25,000 Da were used. Brines with different salinity were prepared using a mixture of the standard brine components (2.0% KCl + 5.5% NaCl + 0.45% MgCl_2_ + 0.55% CaCl_2_), and the gelation performance of the HPAM/PEI system (1.0 wt.% HPAM + 0.4 wt.% PEI) in these brines at 60 °C was investigated first; see Table 8.

As a general trend, with increasing salinity as well as ionic strength, both the viscosity of the gelant and the gel strength decreased, and the gelation time was significantly prolonged. Notably, even in brines with a salinity of 80,000 mg·L^−1^, the HPAM/PEI system with 25,000 Da PEI still achieved grade D gel strength, albeit with a prolonged gelation time of 23 days. The delayed crosslinking effect induced by the salt was primarily due to its influence on the conformation of the HPAM molecules in the solution. The cations in the salt migrate to the hydrolyzed Stern layer of the HPAM molecule, leading to the neutralization of some of the negative charges on the polymer molecular chain [44]. This, in turn, reduces the electrical potential; causes the HPAM molecular chain to curl and shrink, reducing the hydrodynamic radius [45]; and partially hides some amide groups, rendering contact with the amine groups of PEI more difficult. This, known as the charge-shielding effect, becomes more pronounced with increasing ionic strength, as evidenced by the decreasing initial viscosity of the gelant with increasing ionic strength. The charge-shielding effect of salts is also reflected in the reduced ability of carboxylate ions to form hydrogen bonds with water, leading to a decrease in their hydrolysis degree and the weakening of the intramolecular chain repulsion. In addition, certain cations present in the salt, particularly divalent cations such as Ca^2+^ and Mg^2+^, can induce the flocculation and sedimentation of HPAM under specific conditions. To further elucidate the effects of key cations such as Na^+^, K^+^, and Ca^2+^ on the gelation performance of the HPAM/PEI system, we conducted gelation experiments in single-salt solutions.

**Figure 10:**
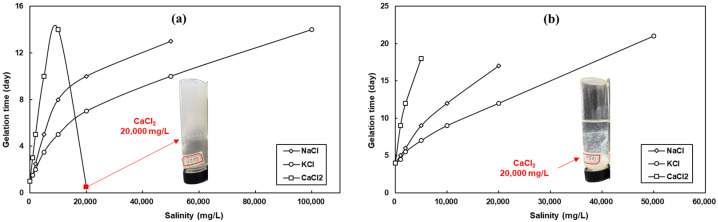
Effect of individual salt concentrations on gelation time: (**a**) 1.0 wt.% HPAM + 0.4 wt.% PEI; (**b**) 0.4 wt.% HPAM + 0.16 wt.% PEI.

**Figure 11:**
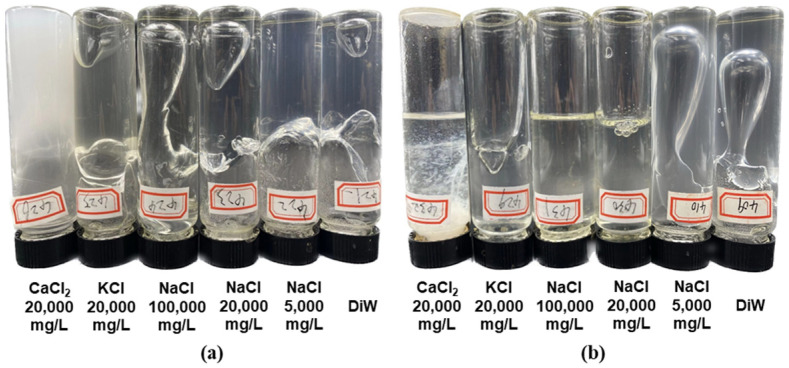
Effect of various salts on strength of mature gel: (**a**) 1.0 wt.% HPAM + 0.4 wt.% PEI, and (**b**) 0.4 wt.% HPAM + 0.16 wt.% PEI.

Figure 10 and Figure 11 depict a trend that is consistent with the results obtained with the mixed brine. The gelation time of the HPAM/PEI system increased while the gel strength decreased with the increasing single-salt concentration. Lower-concentration systems were significantly less tolerant to salt at equivalent salinity. For instance, the system with 1.0 wt.% HPAM in 2.0% KCl or NaCl was able to achieve grade F gel strength, while the system with 0.4 wt.% HPAM only achieved a grade D. There are many well-established methods for the improvement of the salt resistance of gels, such as introducing chelating agents or modifying the polymers [46,47,48], and we hope to optimize these methods in our future work.

The delayed crosslinking effect of cations can be ranked as Ca^2+^ > Na^+^ > K^+^. The delayed crosslinking was due to the salt compression of the HPAM molecular conformation, as mentioned above, while the variance in the delay was due to the different charge densities of the cations (ionic charge/size) [49]. Although Na^+^ has the same charge as K^+^, its smaller radius results in a higher charge density compared to K^+^. As a result, Na^+^ exerts a stronger charge-shielding effect on the HPAM molecular chains, resulting in a longer gelation time and weaker gel strength. This pattern was more pronounced in the system with 0.4 wt.% HPAM; see Figure 11b. Being divalent, Ca^2+^ has nearly twice the charge density and four times the ionic strength of Na^+^ and K^+^, resulting in the strongest delayed crosslinking effect. In the system with 1.0 wt.% in 2.0% CaCl_2_, rapid gelation occurred, resulting in a milky white gel appearance; see Figure 11a. This rapid gelation was mainly due to electrostatic ionic bonding between the carboxyl group on HPAM and Ca^2+^. This type of crosslinking exhibits a fast reaction rate and is difficult to control. In the system with 0.4 wt.% HPAM, Ca^2+^ electrostatically flocculated with the HPAM only locally due to the low concentration of HPAM; see Figure 11b.

#### 3.2.3. pH

The gel system was the same as in the previous section, and the brine had a salinity of 5000 mg·L^−1^ at 60 °C.

**Figure 12:**
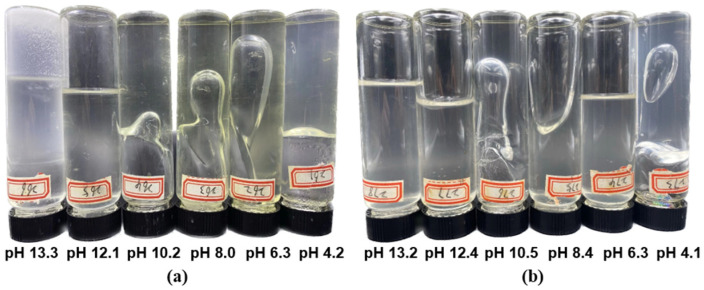
Matured gels at different pH: (**a**) 1.0 wt.% HPAM + 0.4 wt.% PEI; (**b**) 0.4 wt.% HPAM + 0.16 wt.% PEI.

**Figure 13:**
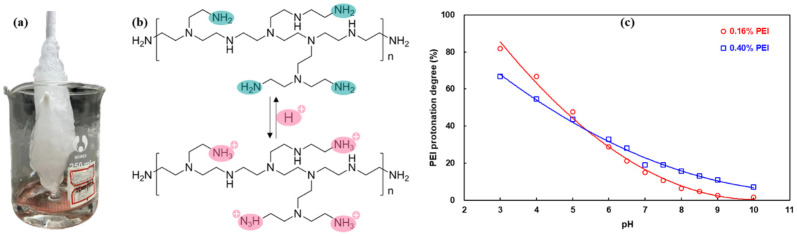
(**a**) Gelation during stirring; (**b**) amine protonation on PEI; (**c**) PEI protonation degree versus pH.

The same trends were observed regarding the effect of the pH on the gelation time and gel strength in both gel systems, see Table 9. The most favorable conditions for the HPAM/PEI gel system were the neutral and alkaline conditions, with the best results obtained at pH~10.5. However, it failed to gel under highly alkaline conditions (pH~13). The 0.4 wt.% HPAM system failed to gel at weakly acidic conditions (pH = 6), but the gel strength of the 1.0 wt.% HPAM system reached grade F; see Figure 12. Notably, both gel systems exhibited rapid gelation at pH = 4. The high-concentration gel system initiated crosslinking even during stirring (Figure 13a) and achieved grade H gel strength.

The pH has a significant effect on the state of both the HPAM and PEI in the solution, with PEI being more sensitive, often referred to as a “proton sponge” [42]. As the pH decreases, some of the amine groups on the PEI molecule become protonated. This process is reversible [18], as shown in Figure 13b. The degree of protonation of PEI decreases with increasing pH; see Figure 13c. Moreover, PEI solutions tend to be alkaline and possess a strong buffering capacity, which becomes even more pronounced at higher concentrations. For this reason, for the HPAM/PEI system, the mixture of PEI with HPAM can be kept in an alkaline condition (pH 9.5~10.5). On the other hand, both acidic and alkaline conditions accelerate the hydrolysis of HPAM [50]. 

Based on the experimental results and the above analysis, the most favorable pH environment for an HPAM/PEI gel system is alkaline (pH~10.5), followed by weakly alkaline and neutral environments. Under alkaline conditions (pH~10.5), most of the amines are not protonated and the carboxylic acid groups are deprotonated, meaning that the amine lone pair in the primary and secondary amines of PEI can complex with the deprotonated carboxylic acid group to create an electrostatic interaction that leads to gelation. The gelation performance of the HPAM/PEI system deteriorated under neutral conditions, which may be attributed to the fact that HPAM is least hydrolyzed under these conditions, and the charge-shielding effect of excess NaOH may be responsible for the failure of gelation under strong alkaline conditions.

The rapid gelation observed at pH~4 in both systems is attributed to the rapid crosslinking reaction between HPAM and highly protonated PEI. Al-Muntasheri and Hardy also reported a similarly rapid gelation process in a PAtBA/PEI gel system at pH~3.5 [51,52], although the mechanism of this crosslinking was not clear. However, it may play a role in the gelation of HPAM/PEI systems under specific reservoir conditions, such as those involving CO_2_ injection. Understanding the extent to which this crosslinking reaction contributes to the gelation process and its controllability could further broaden the application of the HPAM/PEI system.

### 3.3. Plugging Performance

**Figure 14:**
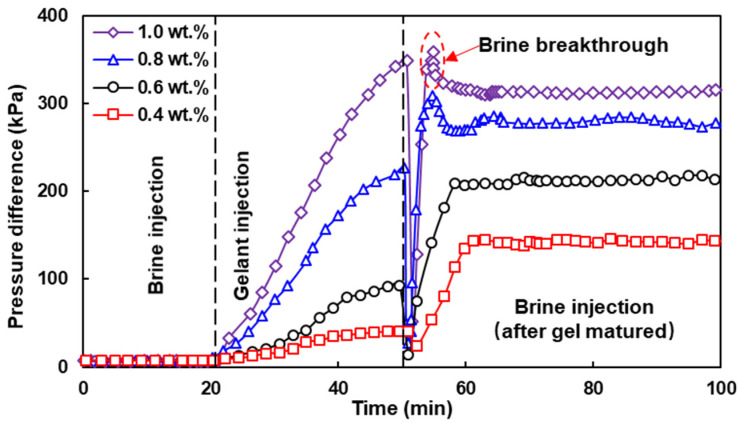
Comprehensive curves of plugging process in HPAM/PEI systems.

Based on the gelation performance, this experiment evaluated the plugging performance of the HPAM/PEI system at 60 °C in low-permeability cores with a through-type fracture. The PEI-to-HPAM concentration ratio was fixed at 2:5 and the pH of all gelants was adjusted to approximately 10.5. The highest concentration of HPAM was set to 1.0 wt.%, which already achieved a grade H gel strength. The salinity of the brine was 5000 mg·L^−1^, which is close to the salinity of the formation water of Nanniwan, a low-permeability reservoir in the Ordos Basin, China. The brine and gelant were injected at a rate of 1 mL·min^−1^. The integrated curve representing the plugging and water-driven processes is shown in Figure 14. The curves were aligned to the start time of each process, with the exception of the gelation process. The residual resistance factor (Frr) is an indicator of the ability of the gel to reduce the permeability of the fracture and was obtained from the ratio of the permeability measured by the brine before and after plugging.

As the concentration of the gel system increases, the brine breakthrough on the pressure curve becomes more pronounced (Figure 14) and the residual resistance factor (Frr) increases significantly. The stabilized pressure gradient (525.60 kPa·m^−1^) after plugging the system with 0.1 wt.% HPAM exceeded the threshold pressure gradient (470 kPa·m^−1^) of the core matrix; see Table 10. The blocking of the fractured core was achieved. On the other hand, as the HPAM concentration increased, the pressure of the gel injection process also increased. When the HPAM concentration was 1.0 wt.%, the maximum injection pressure gradient during the gel solution injection process exceeded the threshold pressure gradient of the core matrix, which could lead to the gelant penetrating into the rock matrix, resulting in a decrease in its permeability, which is unfavorable for hydrocarbon production.

In low-permeability reservoirs, lower injection pressure is an important requirement to ensure smooth gelant injection into the formation and flow channel blocking, especially in reservoirs with deep flood channels. Therefore, it is of great importance to further improve the fracture plugging effect of the low-concentration HPAM/PEI system to popularize the application of this system in fractured low-permeability reservoirs. Here, we introduced a multi-slug plugging method optimized for the HPAM/CrAc system in our previous work [35] for a system with an HPAM concentration of 0.6 wt.%. In particular, the first slug was 0.5 PV and the second and third slugs were 0.25 PV, respectively, and the later slugs were injected after the former slugs were sufficiently gelatinized. The experimental results are shown in Table 11.

The results show that the multi-slug plugging method increased the residual resistance factor of the gel system with 0.6 wt.% HPAM to 42.30, which corresponds to a subsequent water-driven pressure gradient of 516.75 kPa·m^−1^, and achieved the blocking of the fractured cores. The maximum pressure gradient during the multi-slug injection of the gelant also did not exceed the threshold pressure gradient of the core matrix. Furthermore, more stable plugging was achieved, resulting in a reduction in Frr of less than 3%, even after 500 PV of brine flushing. In contrast, the reduction in Frr for single-slug plugging was more than 18%.

**Figure 15:**
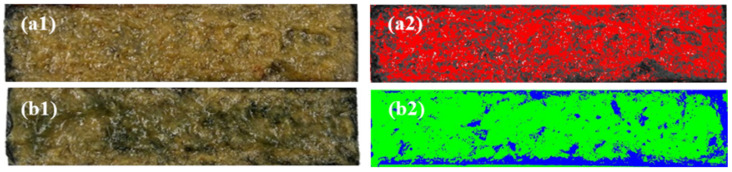
Gel distribution and water flooding traces within fractures after water flooding: (**a1**,**a2**) multi-stage plugs; (**b1**,**b2**) single-stage plugs.

Using the Image J software (1.51i) and the average fluorescence intensity monitoring method, images taken of the same portion inside the fracture after brine flooding were processed to extract the gel distribution and water flooding trace. The left side showed the original image after opening the fracture, while the right side showed the processed image; see Figure 15. In processed image, the red and green color represent the distribution of the HPAM/PEI gel system, the grayish black and blue color represents the water flooding trace. After brine injection, the gel distribution corresponding to the multi-slug process accounted for 57.97% of the fracture area, while the single-slug process corresponded to only 35.14%. The step-by-step compaction produced by the multi-slug injection of the gelant enhances the bonding effect between the gel and the fracture wall [35,53], which is the intrinsic reason for its better plugging performance and scour resistance.

The above results confirm that the multi-slug plugging method is an effective means for improving the plugging efficiency of a low-concentration HPAM/PEI system. Later work can be carried out according to the specific reservoir environment, using the low-concentration HPAM/PEI system to optimize the plugging process and achieve a better water plugging effect.

## 4. Further Research

**Figure 16:**
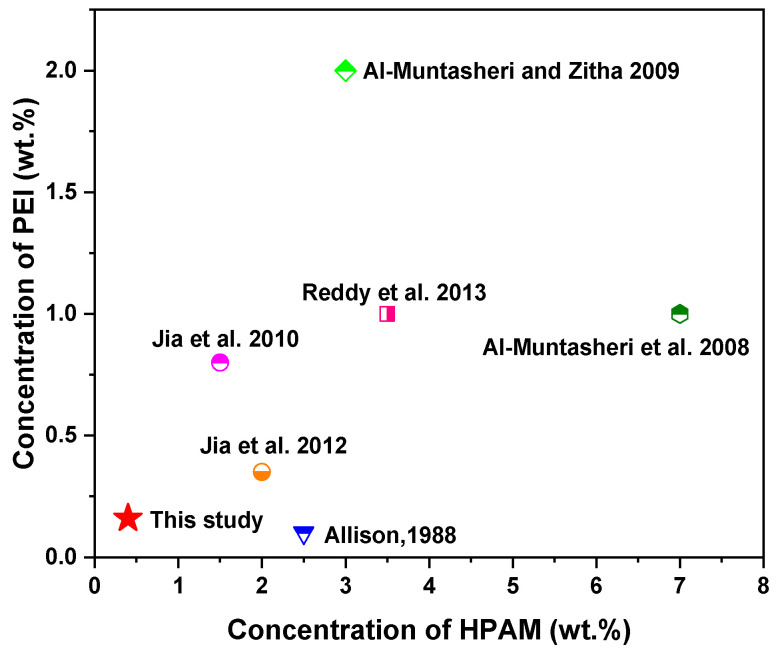
Concentration comparison of the HPAM/PEI system with those of previous studies [17,18,20,54,55,56].

Compared with previous studies, our work investigated the gelation performance of the HPAM/PEI system at a very low concentration (0.4 wt.% HPAM + 0.16 wt.% PEI); see Figure 16. The plugging performance results demonstrate the effectiveness of the low-concentration HPAM/PEI gel system in plugging fractures in low-permeability reservoirs. These promising results indicate the great potential for its widespread application in conformance control and water shutoff practices in low-permeability reservoirs.

In the future, methods to improve the crosslinking efficiency of low-concentration HPAM/PEI gel systems, such as the optimization of the PEI activators and the introduction of crosslinking agents that can crosslink with the carboxyl groups of HPAM to achieve multiple crosslinking, can also be further explored to improve the gel strength. It is also worth investigating the preferred chelating agents to improve the salt resistance of the HPAM/PEI system.

## 5. Conclusions

1. The gelation time of the HPAM/PEI system with an HPAM concentration ranging from 0.4 to 2.0 wt.% varies from less than 1 h to 23 days, and the highest gel strength that can be achieved is grade H. The appropriate ratio of the PEI-provided imine groups and HPAM-provided amide groups is approximately 1.6:1~5:1. Moreover, changes in the HPAM structure and concentration have a more significant effect on the gelation performance than changes in those of PEI.
2. Increasing the hydrodynamic radius of HPAM, which is determined by both the molecular weight and the hydrolysis degree, can directly improve the gelation performance of the HPAM/PEI system and reduce the minimum critical concentration of HPAM. The gelation performance associated with branched PEI was significantly better than that of linear PEI, and, for branched PEI, the macroscopic gelation performance of the HPAM/PEI gel system could only be improved by substantially increasing the molecular weight.
3. Higher temperatures promoted the gelation performance considerably, related to their effect on the structure of HPAM. The order of the delayed crosslinking effect of cations on the gel system was Ca^2+^ > Na^+^ > K^+^. The pH controlled the crosslinking reaction, primarily due to the protonation degree of PEI and hydrolysis degree of HPAM, and the ideal gelation environment for the HPAM/PEI system fell within the alkaline state (pH~10.5).
4. The low concentration of the HPAM/PEI system can realize the effective blocking of through-type fractures in low-permeability reservoirs after gelation. The multi-slug method could further improve the plugging ability and stability of the HPAM/PEI system; on the other hand, the method could further reduce the concentration of the HPAM/PEI system used, which is favorable for its widespread application in low-permeability reservoirs.

## Figures and Tables

**Table 1:** Gelation performance parameters with HPAM with different molecular weights.

Mw of HPAM (×10^6^ Da)	Hydrolysis Degree (%)	Hydrodynamic Radius (nm)	Temperature (°C)	Gelation Time (Day)	Strength Code
5	9.62	297.12	60	2.0	H
7	5.47	123.51		4.0	F
10	6.12	205.88		3.5	E
12	7.64	360.30		0.5	H
14	3.75	91.96		/	No gelation (within 30 days)
5	9.62	183.90	40	5.0	G
7	5.47	77.52		9.0	E
10	6.12	131.89		8.0	D
12	7.64	228.43		1.0	H
14	3.75	59.46		/	No gelation (within 30 days)

**Table 2:** Parameters of gelation performance with different PEI structures.

Temperature (°C)	Molecular Weight of PEI (Da)	Branching Degree (%)	Gelation Time (Day)	Strength Code
60	600	52	2.0	H
	1800	45	2.0	H
	10,000	60	1.0	H
	25,000	48	0.5	H
	25,000	0	10.0	C
40	600	52	3.0	G
	1800	45	3.0	G
	10,000	60	1.5	H
	25,000	48	1.0	H
	25,000	0	15.0	B

**Table 3:** Relative mass ratio of amino nitrogen on PEI (25,000 Da).

PEI Type	Molecular Weight of Repeating Unit (g/mol)	Number of Repeating Units	Nitrogen in Primary Amine (%)	Nitrogen in Secondary Amine (%)	Nitrogen in Tertiary Amine (%)
L-PEI	43	580	0.11	32.48	0
B-PEI	416	60	13.55	6.72	13.44

**Table 4:** Parameters of gelation process with varying HPAM concentrations.

Temperature (°C)	HPAM Concentration (wt.%)	2.0	1.6	1.2	0.8	0.4
60	Gelant viscosity (mPa·s)	364.3	274.3	123.4	76.2	24.8
	Gelation time (day)	0.5	1	1.5	2.5	4
	Strength code	H	H	H	G	E
40	Gelant viscosity (mPa·s)	391.2	305.5	141.5	84.4	27.3
	Gelation time (day)	1	2	2.5	4	7
	Strength code	H	H	G	F	D

**Table 5:** Critical overlap concentration (C*) and hydrodynamic radius of HPAM.

Molecular Weight (×10^6^ Da)	5	7	10	12	14
Hydrolysis degree (%)	9.62	5.47	6.12	7.64	3.75
C* (mg·L^−1^)	5033.05	8197.31	6523.26	3836.95	8215.13
Hydrodynamic radius (nm, 60 °C)	297.1	123.5	205.8	360.3	91.6

**Table 6:** Parameters of the gelation process with different PEI concentrations.

PEI Concentration (wt.%)		0.6	0.5	0.4	0.3	0.2	0.1	0.05
25,000	Gelant viscosity (mPa·s)	114.4						
	Gelation time (day)	1	1	1	1.5	2	3.5	7
	Strength code	H	H	H	G	G	G	D
1800	Gelant viscosity (mPa·s)	86.2						
	Gelation time (day)	1	1	1.5	2	2.5	4.5	No gelation
	Strength code	H	H	G	G	G	E	

**Table 7:** Parameters of gelation performance at different temperatures.

System Number	System	Temperature (°C)	Gelation Time (Day)	Strength Code	Ea (kJ·mol^−1^)
1	1.0 wt.% HPAM + 0.1 wt.% PEI	20	21	E	36.95
		40	8	F	
		60	3.5	G	
		80	1.5	H	
2	0.9 wt.% HPAM + 0.2 wt.% PEI	20	16	D	39.07
		40	4	F	
		60	2	G	
		80	1	G	
3	0.8 wt.% HPAM + 0.4 wt.% PEI	20	12	D	46.60
		40	3.5	E	
		60	1	F	
		80	0.5	G	

**Table 8:** Parameters of gelation process in standardized brines with different salinity.

Salinity (mg·L^−1^)	Ionic Strength (mol/L)	Gelant Viscosity (mPa·s)	Gelation Time (Day)	Strength Code
Deionized water	/	114	1.0	H
4000	1.5	87	3.0	G
8000	3.0	71	4.5	F
20,000	7.5	54	7.0	E
40,000	15.0	48	10.0	E
60,000	22.5	36	16.0	D
80,000	30.0	29	23.0	D

**Table 9:** Gelation time and strength of HPAM/PEI gel systems at different pH levels.

Gel Systems	pH	Gelation Time (Day)	Strength Code
1.0 wt.% HPAM + 0.4 wt.% PEI	4.2	<1 h	I
	6.3	17	F
	8.0	5	G
	10.2	1	H
	12.1	/	No gelation
	13.3	/	No gelation
0.4 wt.% HPAM + 0.16 wt.% PEI	4.1	<1 h	H
	6.3	/	No gelation
	8.4	8	E
	10.5	4	F
	12.4	20	C
	13.2	/	No gelation

**Table 10:** Plugging performance parameters in fractured cores.

HPAM Concentration (wt.%)	PD * of Brine Injection (kPa)	Permeability (D)	PD_max_ of Gelation Injection (kPa·m^−1^)	PD of Brine Injection after Plugging (kPa·m^−1^)	Frr
0.4	7.62	15.26	68.11	236.90	18.65
0.6	7.43	15.65	154.06	352.87	28.50
0.8	7.05	16.49	377.86	460.40	39.18
1.0	6.81	17.07	581.6	525.60	46.30

* PD: pressure gradient.

**Table 11:** Fractured core plugging data with different plugging processes.

Plugging Method	PD_max_ of Gelation Injection (kPa·m^−1^)	Frr 50 PV	Frr 100 PV	Frr 200 PV	Frr 500 PV	Percentage of Gel Distribution (%)
Single slug	154.06	28.50	26.42	25.10	23.30	57.97
Three slugs	274.22	42.30	42.05	41.56	41.09	35.14

## Data Availability

Data are contained within the article.

## Abbreviations and Symbols

Abbreviations	Name	
HPAM	Partially hydrolyzed polyacrylamide	
PEI	Polyethyleneimine	
L-PEI	Linear polyethyleneimine	
B-PEI	Branched polyethyleneimine	
PAtBA	Polyacrylamide tertiary butyl acrylate	
CrAc	Chromium acetate	
Symbols	Name	Unit
*HD*	Hydrolysis degree	%
*BD*	Branching degree	%
*R_h_*	Hydrodynamic radius	nm
Mw	Molecular weight	Da (Dalton)
C*	Critical overlap concentration	mg·L^−1^
*Ea*	Activation energy	kJ·mol^−1^
*GT*	Gelation time	h or day
*PD*	Pressure gradient	kPa·m^−1^
PV	Pore volume	Dimensionless
Frr	Residual resistance factor	Dimensionless
